# Measurement of effective renal plasma flow using model analysis of dynamic CT in the preoperative evaluation of the renal transplant donors

**DOI:** 10.20407/fmj.2018-017

**Published:** 2020-02-11

**Authors:** Yumi Kataoka, Hitoshi Nishio, Ryo Matsukiyo, Ryoichi Kato, Midori Hasegawa, Takashi Kenmochi, Ryoichi Shiroki, Hiroshi Toyama, Takashi Ichihara, Shigeki Kobayashi

**Affiliations:** 1 Faculty of Medical Quantum Science, Fujita Health University Graduate School of Health Sciences, Toyoake, Aichi, Japan; 2 Department of Radiology, Fujita Health University Hospital, Toyoake, Aichi, Japan; 3 Department of Nephrology, Fujita Health University, School of Medicine, Toyoake, Aichi, Japan; 4 Department of Organ Transplant Surgery, Fujita Health University, School of Medicine, Toyoake, Aichi, Japan; 5 Department of Urology, Fujita Health University, School of Medicine, Toyoake, Aichi, Japan; 6 Department of Radiology, Fujita Health University, School of Medicine, Toyoake, Aichi, Japan; 7 Department of Artifical Intelligence in Medical Imaging Development, Fujita Health University, School of Medicine, Toyoake, Aichi, Japan; 8 Faculty of Radiological Technology, Fujita Health University, School of Medical Sciences, Toyoake, Aichi, Japan

**Keywords:** Low radiation dose CT, Dynamic CT, Effective renal plasma flow

## Abstract

**Objectives:**

Renal scintigraphy is widely used to evaluate residual function of a transplanted kidney from the donor. Dynamic computed tomography (CT) imaging can evaluate both kidney morphology and regional renal function. The aim of this study was to develop an imaging protocol and a calculation method using dynamic CT for assessing the effective renal plasma flow (ERPF) by model analysis, and to evaluate the validity of the obtained ERPF values.

**Methods:**

Preoperative dynamic CT examination with a low radiation dose exposure system was performed for 25 renal transplant donors, and ERPF was calculated from the obtained images (CT-ERPF). To calculate CT-ERPF, we set the region of interest (ROI) in the renal cortex using automatic ROI-setting software developed in our laboratory. We compared the processing time with automatic and manual ROI settings. To evaluate the validity of CT-ERPF, we examined the correlation of age with CT-ERPF and compared with reported ERPF values. We also compared the uptake rates of technetium-99m-dimercaptosuccinic acid and CT-ERPF in terms of the right-to-left ratio.

**Results:**

There was good agreement of CT-ERPF assessed using automatic and manual ROIs. CT-ERPF was negatively correlated with age and showed values below the reference ERPF range in 21 cases. The right-to-left ratio of CT-ERPF showed a significant correlation with that of technetium-99m-dimercaptosuccinic acid.

**Conclusions:**

Using our method, CT-ERPF was a useful indicator for preoperative evaluation of donor’s renal function.

## Introduction

Measurement of residual renal function of the donor kidney is necessary for transplantation surgery. Renal function can be evaluated preoperatively by several methods, including creatinine clearance, estimated glomerular filtration rate (eGFR), and renal scintigraphy. Morphological evaluation is also performed using dynamic contrast-enhanced computed tomography (dynamic-CT) with iodine contrast medium. In our hospital, three-phase dynamic CT is performed for patients with renal disease, and includes pre-contrast of the first phase, the nephrographic phase, and the excretory phase. Frennby et al.^1^ reported that iodinated contrast medium had excellent properties as a marker of GFR.

A focus of our laboratory is the development of techniques for calculating renal plasma flow from dynamic CT with model analysis. In previous studies, renal blood flow (RBF) was accurately calculated using the deconvolution method with dynamic CT imaging.^2^ This method requires dynamic CT imaging using 20 scans of the first-pass image with one bolus injection of contrast medium. As the area under the curve of the input/output function must be accurately calculated, it is necessary to satisfy the sampling conditions for correct capture of the shape of the time attenuation curve (TDC) at the interval and the number of dynamic scans. Therefore, if this method is applied as used in 320-row multidetector row CT, the exposure radiation dose may be markedly increased. Appropriate minimization of radiation exposure by decreasing the number of scans is important. The purpose of this study was to develop an imaging protocol and calculation method using dynamic CT for calculation of effective renal plasma flow (ERPF) by model analysis, and to evaluate the validity of the obtained ERPF values.

## Methods

This study was approved by the Health Science Ethics Committee at Fujita Health University and was conducted in accordance with the Declaration of Helsinki and Good Clinical Practice. Written informed consent was obtained from all study subjects. Dynamic CT examination (Aquilion ONE; Canon Medical Systems, Tokyo, Japan) with addition of low radiation dose was performed for 25 renal transplant donors (nine men, sixteen women, 37–73 years old, average age of 58.1 years), and ERPF measurements were performed from the obtained images (CT-ERPF). Contrast medium was injected using a dual-shot GX 7 (Nemotokyorindo, Tokyo, Japan). Data analysis was performed using MATLAB R2016a (MathWorks, Natick, MA, USA) for numerical analysis programming language and OsiriX version 7.0.4 (OsiriX Foundation, Geneva, Switzerland) for medical image management software.

### Kinetic model of iodine contrast medium in kidney

**Figure 1:**
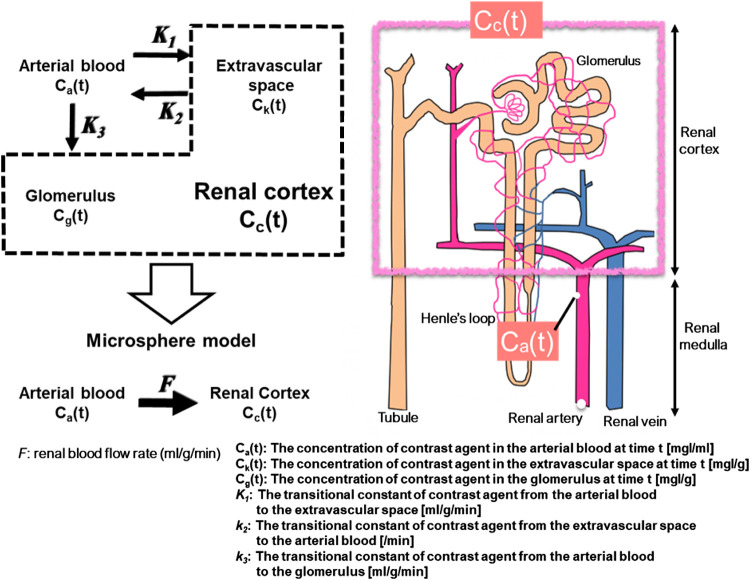
Microsphere model. C_c_(t) is the concentration of contrast medium in the renal cortex at time t. F (ml/g/min) is the renal blood flow rate. As time is required for the contrast medium to pass through the glomerulus in the initial state after administration, the contrast medium seems to accumulate in the glomerulus. Thus, a microsphere model can be assumed.

In this study, the following pharmacokinetic model of iodine contrast medium in the body was used for analysis. The contrast medium administered intravenously flows from the renal artery to the glomerulus. Contrast medium filtered by the glomerulus flows to the renal tubule, while contrast medium that has not been filtered flows to the renal vein. The kidney comprises the renal cortex and renal medulla, with renal tissue, glomeruli, and tubules in the kidney cortex, and only renal tissue in the renal medulla. The concentration of contrast medium in the renal cortex at time t (C_c_(t)) can be described by Equation 1:

(1) $$C_{c}\text{(}t\text{)}=C_{k}\text{(}t\text{)}+C_{g}\text{(}t\text{)}\text{,}$$

where C_k_(t) (mg I/g) is the concentration of contrast medium in kidney tissue, and C_g_(t) (mg I/g) is the concentration of contrast medium in glomeruli. As time is required for the contrast medium to pass through the glomerulus in the initial state after contrast administration, the contrast medium appears to accumulate in the glomeruli. Thus, a microsphere model (Figure 1) can be assumed.

In Figure 1, F (ml/g/min) reflects the renal blood flow rate. At this time, contrast medium concentration in the kidney cortex at time t (C_c_(t)) is expressed by the amount of contrast medium that continues to accumulate in the renal cortex from the arterial blood, as shown in Equation 2:

(2) $$C_{c}\text{(}t\text{)}=F\int_{0}^{t}C_{a}\text{(}\tau \text{)}d\tau \text{,}$$

where C_a_(t) (mg I/ml) is the concentration of contrast medium in the arterial blood at time t. From Equation 2, (X(t) and Y(t)) are plotted to obtain an approximate straight line as $X\text{(}t\text{)}=\int_{0}^{t}C_{a}\text{(}\tau \text{)}d\tau$, $Y\text{(}t\text{)}=C_{c}\text{(}t\text{)}$. F can be calculated as the slope obtained from the approximate straight line (Patlak plot method).^3,4^

### Hematocrit correction

To calculate ERPF, hematocrit correction must be performed for renal blood flow F calculated from Equation 2. Solving Equation 2 for F yields Equation 3:

(3) $$F=\frac{C_{c}\text{(}t\text{)}}{\int_{0}^{t}C_{a}\text{(}\tau \text{)}d\tau }\text{,}$$

where C_a_(t) (mg I/ml) is the concentration of contrast medium in the arterial blood of the renal artery. Because the concentration of contrast medium and the CT value have a proportional relationship, C_a_(t) is represented by the CT attenuation value (HU). Pixels in the arterial blood obtained from the CT image have units of HU/voxel, with one voxel represented by one pixel. The contrast medium exists in the plasma, and to obtain C_a_(t) from this CT pixel, plasma volume V (ml) of the voxel constituting this pixel must be converted to the contrast medium concentration. This plasma volume is calculated using the hematocrit. The renal artery is a large blood vessel corrected for the hematocrit value of HCT_LV_ in a large blood vessel. The amount of plasma contained in the voxel of one pixel of the renal artery is $\text{(}1-{\text{HCT}}_{\text{LV}}\text{)}\cdot V$ (ml), and the concentration of contrast medium contained in the plasma within this voxel is $\frac{C_{a}\text{(}t\text{)}}{\text{(}1-{\text{HCT}}_{\text{LV}}\text{)}\cdot V}$ (HU/voxel/ml).

C_c_(t) is the concentration of contrast medium in the kidney cortex, and represents the amount of contrast medium per unit weight. However, the units of C_c_(t) obtained from the CT image are CT value per voxel (HU/voxel). First, the units of C_c_(t) must be converted from CT value per voxel to CT value per unit weight. When the density of renal tissue is assumed to be ρ (g/ml), the weight can be obtained by multiplying the voxel by the density of the kidney tissue. Therefore, the CT attenuation value per 1 g of kidney tissue is expressed as (HU·ml/voxel/g).

Next, because glomerular capillaries are contained in the kidney cortex, hematocrit correction is required as for C_a_(t). Because the glomerular capillary is a small blood vessel, the value is corrected for the hematocrit in a small blood vessel, HCT_SV_. The amount of plasma contained in the voxel represented by one pixel of kidney cortex is therefore $\text{(}1-{\text{HCT}}_{\text{SV}}\text{)}\cdot V$ (ml). The concentration of contrast medium contained in the plasma within this voxel is expressed as $\frac{C_{a}\text{(}t\text{)}}{\text{(}1-{\text{HCT}}_{\text{LV}}\text{)}\cdot \rho \cdot V}$ (HU/voxel/g).

ERPF corrects for the hematocrit in large and small blood vessels, and the correction for kidney tissue density is shown in Equation 4:

(4) $$\text{ERPF}=\frac{\text{(}1-{\text{HCT}}_{\text{LV}}\text{)}}{\text{(}1-{\text{HCT}}_{\text{SV}}\text{)}\cdot \rho }\,\frac{C_{c}\text{(}t\text{)}}{\int_{0}^{t}C_{a}\text{(}\tau \text{)}d\tau }\text{.}$$

Substituting Equation 3 into Equation 4 yields Equation 5, which converts renal blood flow F to ERPF:

(5) $$\text{ERPF}=[\frac{\text{(}1-{\text{HCT}}_{\text{LV}}\text{)}}{\text{(}1-{\text{HCT}}_{\text{SV}}\text{)}\cdot \rho }]\cdot F\text{.}$$

For this study, we performed hematocrit corrections using HCT_LV_=0.45 and HCT_SV_=0.25.^5^

### CT imaging protocol

In the preoperative examination of donors, CT imaging from the liver to the pelvis was performed, with three phases of pre-contrast, nephrogram, and excretion. For analysis of ERPF using the kinetic model (see previous section), dynamic CT imaging was added before the renal artery phase. The study imaging protocol used dynamic CT with a tube voltage of 100 kV, auto exposure control (tube current, 84–135 mA), an X-ray rotation speed of 0.5 s/rotation, and a 2.0-s interval for 20 s. Imaging was started 8 s after starting injection. Contrast medium was injected at 600 mg I per kg body weight within 25 s. The infusion rate was approximately 3.3–5.0 ml/s, with a boost injection of 30 ml saline. We used FC04 AIDR 3D Strong (Aquilion ONE; Canon Medical Systems) for the reconstruction function. The average dose at this time was 29.7 mSv, and the average dose of dynamic imaging added on this protocol was 4.5 mSv. The contrast medium used was iopamidol 370 (Bayer Pharmaceutical, Osaka, Japan), omnipaque 300 (Daiichi Sankyo Co., Tokyo, Japan), iomepurol 350 (Eisai Co., Tokyo, Japan), or Optirei 240 (Fuji Pharmaceutical, Tokyo, Japan).

### Creation of input function (C_a_(t))

**Figure 2:**
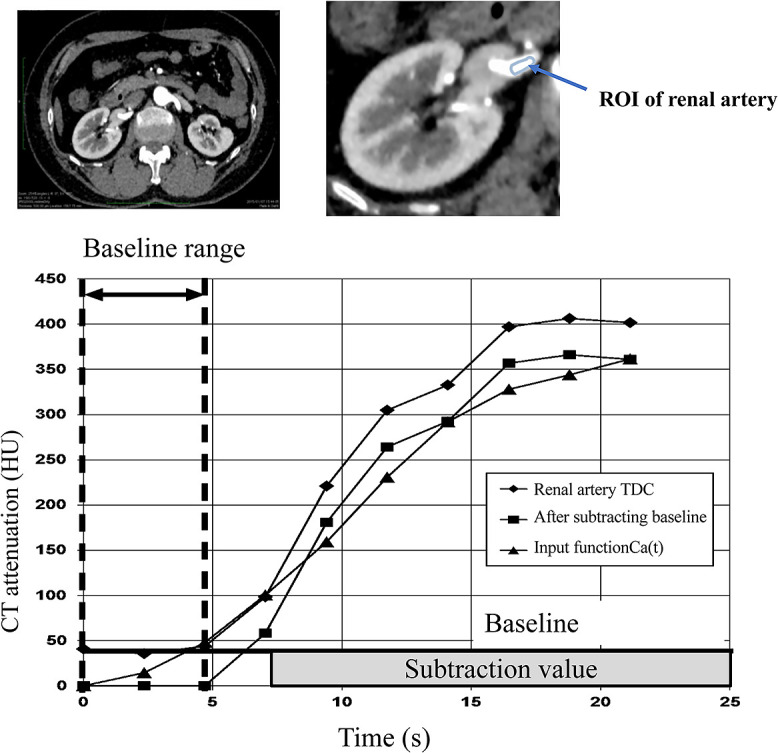
Creation of input function for computed tomography-effective renal plasm flow (CT-ERPF) calculation. A time density curve (TDC) was created from the CT value of the renal artery region of interest (ROI) setting. We visually set the ROI of the renal artery before inflow of contrast medium and used this as the baseline range. CT values of TDC within the pre-contrast range were averaged to create the baseline value. The baseline value was subtracted from the created TDC, and five-point smoothing was performed to obtain the input function of the concentration of contrast medium in the arterial blood at time t (C_a_(t)). CT values of the input function Ca(t) were calculated as $\mathrm{Ca}\text{(}t\text{)}=\sum_{n-2}^{n+2}\text{(}\text{renal artery TDC})/5-\text{baseline value.}$

An ROI was set for each of the right and left renal arteries of the obtained dynamic contrast CT data to generate a TDC (attenuation). The range from the TDC until the CT value started to rise was defined as baseline. An average within the baseline range was calculated as the baseline value, which was subtracted from the TDC. This corrected TDC was processed with five-point smoothing to average the CT values of five points before and after the plot. This value was defined as the input function C_a_(t) (Figure 2). The smoothing processing was important because the original baseline corrected TDC curve can become unstable because of noise. Determining the appropriate baseline value was important for calculating CT-ERPF.

### Creation of output function (C_c_(t))

**Figure 3:**
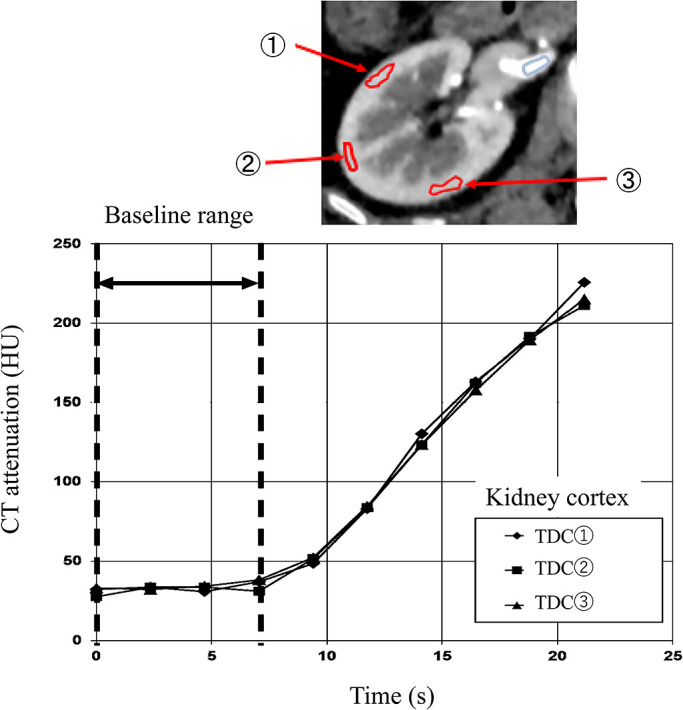
Baseline range setting for the output function used for CT-ERPF calculation. Three pointed ROIs were set in the renal cortex and the TDC was created. The range before the increase in TDC was used as the baseline range, and the average of the three TDCs used as the output function.

In the phase in which the kidney cortex was being enhanced, three ROIs of the kidney cortex were set in the cross-section of the central part of the kidney, and three TDCs were generated from the obtained ROI. The range from the TDC until the CT value started to rise was defined as the baseline range. CT values of the TDC for each pixel within the baseline range of the kidney cortex were averaged to determine the baseline value (Figure 3). Determining this baseline value was also important for calculating CT-ERPF.

### Automatic setting of the renal cortex

The ROI for the renal cortex was automatically set using the following procedure. For removal of bone, the maximum CT value of the first dynamic image after contrast medium administration was calculated, and 10% of the maximum CT value was taken as the threshold value. Pixels with a CT value higher than the threshold value were considered bone regions and removed. For removal of renal artery/renal vein/kidney tissue, a threshold value was obtained using the maximum CT value of the kidney cortex TDC and the CT value in the equilibrium phase. For the CT image in the equilibrium phase, pixels below the threshold value were removed as the renal artery, renal vein, and extrarenal tissue, and both kidneys were extracted. The extracted right and left kidneys were then distinguished based on the left and right renal centroid coordinates. For removal of the renal medulla, in the renal cortex, the CT value in the arterial phase is higher than that in the equilibrium phase, while the converse is true for the renal medulla. Using this relationship, pixels with higher CT values in the equilibrium phase than those in the arterial phase were removed as the renal medulla, allowing the boundary between the kidney cortex and renal medulla to be determined, and the cortical area extracted. The glomerulus may also exist at the boundary between the cortex and medulla. Thus, a process to incorporate the boundary between the cortex and medulla where the glomerulus exists within the cortex was performed. For ROI creation in the renal cortex, the boundary between the extracted renal cortex and renal vein was visually confirmed, and any mislabeled pixels were corrected manually, to create an ROI of the entire renal cortex. An ROI was also created for the entirety of each left and right kidney, and a TDC generated for each pixel. The baseline value was subtracted from this TDC, and as for creation of the input function, five-point smoothing was performed to obtain the output function C_c_(t) (Figure 3).

### Correction of the time difference between input and output functions

The methodology assumes no time lag between input function C_a_(t) calculated from the renal artery and output function C_m_(t) calculated from the renal medulla. However, contrast medium in the renal artery flows into the glomerulus and reaches the renal medulla in approximately 1–2 s. Therefore, to accurately perform model analysis, the time difference between the input function C_a_(t) and renal medullary output function C_m_(t) was calculated and corrected.^6^

For creation of an ERPF image and value for one or both kidneys, renal blood flow F was calculated with the Patlak method (Figures 1–3) using Equation 4. The linear approximation range of the Patlak analysis was performed at three points after the rising edge of the output function C_c_(t). Hematocrit correction was performed on the calculated values for each pixel to prepare an ERPF image. ERPF values of the left and right kidneys, and of both kidneys, were calculated by integrating the ERPF value for each pixel within the ROI set in the renal cortex. For evaluating the accuracy of the automatic ROI of the renal cortex, we compared the processing time for manual and automatic ROIs and the match rate. An ROI of the renal cortex prepared manually and confirmed by radiologists (manual ROI) was evaluated with the following three items:

(1) Match rate between the manual and automatic ROIs. The coincidence rate between the manual and automatic ROIs was calculated using Equation 6:

(6)  (Coincidence rate)=(1–((number of pixels that did not match between manual ROI and automatic
ROI)/(number of pixels rendered as renal cortex by manual ROI)))×100.

(2) Evaluation of CT-ERPF calculated using manual and automatic ROIs. The correlation of CT-ERPFs calculated using manual ROI with automatic ROI was determined by plotting (X, Y) the coordinates (X-axis: CT-ERPF calculated using a manual ROI; Y-axis: CT-ERPF calculated using an automatic ROI) to determine a correlation formula, a correlation coefficient, and a p value.

(3) Comparison of processing time between the manual and automatic ROIs. The manual ROIs were performed in nine of 25 cases under the guidance of a radiologist employed at Fujita Health University Hospital. The required time to set the ROI in the renal cortex was compared between manual and automatic settings in nine cases.

### Evaluation of ERPF calculated by CT

The calculated ERPF was compared with reported reference values.^7–11^ The conventional paraaminohippuric acid clearance (C_PAH_) was defined as the reference ERPF. We also evaluated the age-dependence of ERPF values in our cases. To confirm the validity of CT-ERPF values, the right-to-left ratio of technetium-99m-dimercaptosuccinic acid (^99m^Tc-DMSA) kidney uptake and CT-ERPF values before surgery were evaluated. ^99m^Tc-DMSA scintigraphy was performed in 19 cases, and was performed first on the same day of dynamic CT.

### Statistical analysis

All statistical analyses were performed using statistical software (Matlab R2016a; MathWorks). Statistical comparisons were performed between the CT-ERPF value calculated by automated ROI and the CT-ERPF value calculated by manual ROI, and between the right-to-left ratio of CT-ERPF values and the right-to-left ratio of renal uptake of ^99m^Tc-DMSA. Correlation coefficients of those ratios were obtained, and data analyzed by Pearson’s correlation coefficient. A p-value <0.05 was considered significant. Bland–Altman analysis was performed to compare differences in the ratios. Bias was determined as the average value of differences in CT-ERPF values calculated from both methods, and limits of agreement were set from –1.96 to +1.96 standard deviations of the CT-ERPF value.

## Results

### Evaluation of automatic ROI in the renal cortex

**Figure 4:**
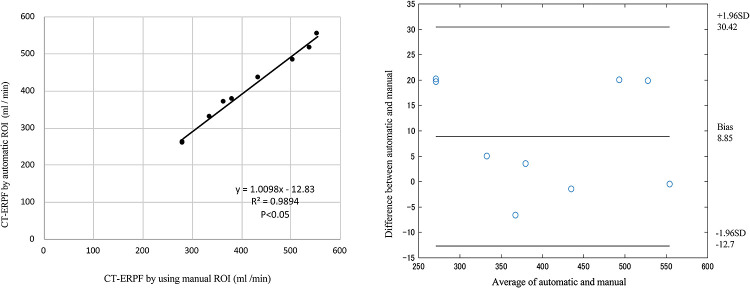
Correlation of CT-ERPFs calculated using manual and automatic ROIs. Left: CT-ERPF values calculated from the manual ROI and the automatic ROI plotted as a correlation. The linear slope is close to 1, with a p value <0.05, indicating a significant positive correlation. Right: Bland–Altman plots showing a bias of 8.85 ml/min and limits of agreement of –12.7–30.42 ml/g/min.

The average agreement rate with the manual ROI before manual correction to the automatic ROI was 81.2%, and the average agreement rate after manual correction was 82.9%. In both kidneys, the linear equation was y=1.010×–12.83 (p<0.05), with a linear relationship close to 0.999 and 1, indicating a significant positive correlation. The correlation of CT-ERPF calculated using the manual and automatic ROIs, and the Bland–Altman analysis, are shown in Figure 4. The bias between ERPF calculated using automatic and manually set ROIs was 8.85 ml/min. The limits of agreement also changed from 30.42 ml/g/min to –12.7 ml/g/min.

### Comparison of processing times for manual and automatic ROI

Processing times for setting ROIs in the renal cortex using manual and automatic methods are shown in Table 1. When all ROIs of the renal cortex were manually set, the average was 16.7 h per case, while the processing time using automatic setting was only 1.36 h on average. Automated ROI processing consisted of both automatic processing and manual correction. The average processing time for automatic processing was 0.0245 h, while the average manual correction processing time was 1.34 h.

### CT-ERPF images and CT-ERPF values

**Figure 5:**
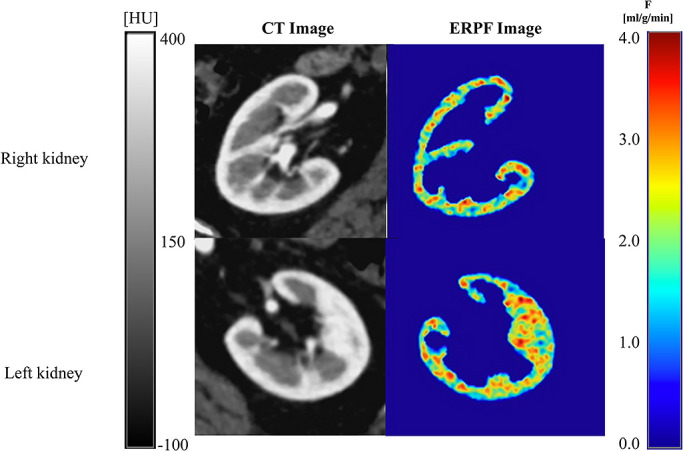
CT image and CT-ERPF image. Left: Contrast-enhanced CT images. Right: CT-ERPF images. The upper image shows the right kidney. The lower image shows the left kidney.

**Figure 6:**
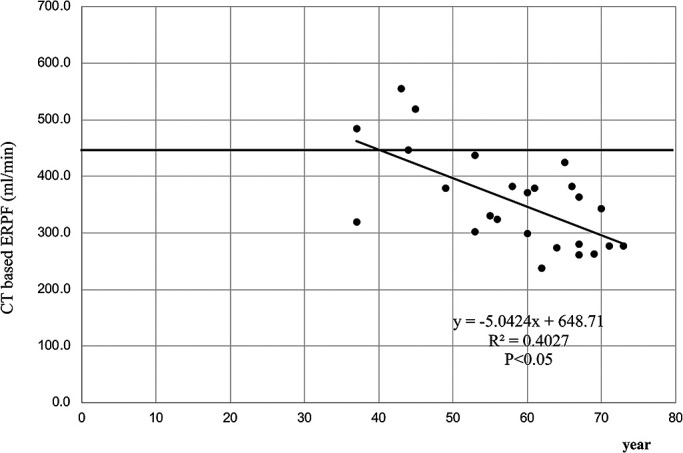
Correlation of age with CT-ERPF in the 25 renal transplant donors. The correlation coefficient R^2^=0.40 (p<0.05) indicates a weak negative correlation. The transverse line shows a lower value of the reference ERPF.

A representative CT-ERPF image is shown in Figure 5. The left panel shows a contrast-enhanced CT image, the right panel the CT-ERPF image, the upper panel shows the right kidney, and the lower panel the left kidney. The calculated CT-ERPFs for both kidneys are shown in Figure 6. Previous non-Japanese studies have reported normal adult conventional ERPFs (C_PAH_) of 442–694 ml/min/1.73 m^2^,^7,8^ while Japanese studies have reported values of 439–817 ml/min/1.73 m^2^.^9–11^ In the present study, all cases were Japanese adult renal transplant donors (men: 37–73 years old; woman: 43–71 years old). Thus, we adopted a reference range of ERPF (C_PAH_) of 439–817 ml/min/1.73 m^2^.

**Figure 7:**
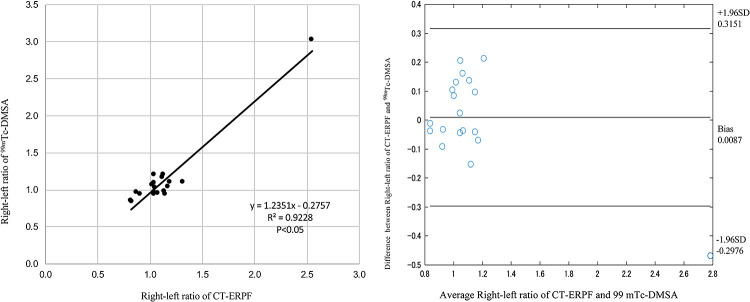
Relationship between CT-ERPF and technetium-99m-dimercaptosuccinic acid (^99m^Tc-DMSA) uptake. Left: Correlation of the right-to-left ratio of CT-ERPF with the right-to-left ratio of ^99m^Tc-DMSA uptake. The correlation coefficient R^2^=0.92 (p<0.05) indicates an excellent positive correlation. Right: Bland–Altman plots showing a bias of 0.0087 and limits of agreement of –0.30–0.32.

Twenty-one cases showed values below the reference range. The results of comparisons of the right-to-left ratio of ^99m^Tc-DMSA kidney uptake with those of CT-ERPF for 19 of the 25 cases are shown in Figure 7. An excellent correlation was observed between ^99m^Tc-DMSA uptake and CT-ERPF. Bland–Altman plots had a bias of 0.0087, and the limits of agreement were –0.30–0.32. One case was outside of this range. In this case, the sizes of the left and right kidneys were significantly different compared with other patients.

## Discussion

### Automatic ROI setting of renal cortex contours

In our method, the total processing time for automatic ROIs was markedly reduced compared with that for manual ROIs. Nevertheless, future studies are required to further reduce the total processing time for routine clinical use.

### Time difference correction between input and output functions

RBF can be accurately calculated by the deconvolution method using TDC.^2^ In this method, dynamic CT imaging requires 20 scans of the first-pass image with one bolus injection of contrast medium. In our proposed model analysis method, dynamic imaging is performed in the initial phase of contrast medium flowing into the cortex, and the input function from the renal artery image and the cortical area are used to calculate the output function for each pixel TDC. A time difference appears in the image because of differences in the flow path of the contrast medium. As the model uses calculations assuming no time difference between the paths, difference correction is necessary.^8^ According to Krier et al.,^12^ output function of the cortex appears as the initial vascular phase, then in the proximal tubule, followed by the distal tubule. To accurately capture the shape of the vascular phase, the latter two phases must be separated in the deconvolution method. Thus, at least two phase TDCs of the vascular and proximal tubules are required. Compared with the deconvolution method, the Patlak method calculates RBF using several points of the initial vascular phase of TDC. Thus, the number of dynamic scans (sampling number) can be reduced.

### Hematocrit correction

Kudo et al. performed hematocrit correction with HCTLV=0.45, HCTSV=0.25, and a brain tissue density of 1.04 g/ml to measure cerebral blood flow.^5^ Thus, in the present study the concentration of contrast medium in the renal cortex was converted from the CT value per voxel to the amount of contrast medium per unit weight,^6^ and the density of renal tissue was the same as that of Kudo et al.^5^ To obtain the ERPF, we used hematocrit correction of large and small blood vessels, and used Equation 7 to consider the density of kidney tissue.

### CT-ERPF compared with reference ERPF

CT-ERPF values using the Patlak method in our study tended to be lower (21 of 25 cases) than reported reference ERPFs. Russell et al. used these measurements interchangeably with the following conversions: ERPF=C_PAH_; clearance of iodine-131-ortho-iodohippurate (C_OIH_)=0.90 C_PAH_; clearance of technetium-99m-mercaptoacetyltriglycine (^99m^Tc-MAG3) (C_MAG3_)=0.59; and C_OIH_=0.53 C_PAH_.^7,8^ We considered that CT-ERPF was not equal to C_PAH_, and that CT-ERPF could be converted by the additional factor. CT-ERPF values decreased with age. Further, Russell et al. reported a decrease in ERPF with aging, which was calculated using the following equation for patients >40 years: ERPF=568–5.83 (age –40)±126.^7,8^ Dujardin et al.^13^ also reported an inverse correlation of age with RBF calculated from contrast MRI data using the deconvolution method. This was because of a moderate age dependence of these values in males. Further, arterial blood flow from Doppler ultrasound measurements tended to decrease with age.^14,15^ We also found a trend towards a decrease in CT-ERPF with age, which may reflect a progressive decrease in renal function.

### CT-ERPF versus ^99m^Tc-DMSA uptake

In the present study, there was an excellent correlation of the right-left ratio of CT-ERPF with that of ^99m^Tc-DMSA uptake. Taylor reported an excellent agreement of ^99m^Tc-DMSA uptake and C_O__I__H_ with serum creatinine ≤2.0 mg/dl,^16^ while Momin et al. reported that ^99m^Tc-DMSA and technetium-99m-diethylenetriaminepentaacetic acid (^99m^Tc-DTPA) scanning methods provided similar relative renal functions values.^17^ Thus, CT-ERPF values may reflect a relative renal function similar to the GFR in the donor’s kidney.

### Study limitations

A limitation of our study is that measurement of RBF (e.g., C_PAH_) was difficult in routine examination in our hospital. For this reason, we performed indirect comparisons and evaluations of the distribution of normal values and nuclear medicine examination findings. Historically, examination by ^99m^Tc-MAG3 is common for renal blood flow examination. However, in our university hospital, the ^99m^Tc-DMSA kidney uptake test was routinely performed for split renal function. Another limitation is that GFR (e.g., eGFR, 24 h creatinine clearance, ^99m^Tc-DTPA scan) should be used to evaluate renal function. Our present data are preliminary findings, and simultaneous measurements of CT-ERPF and CT-GFR are in progress in our laboratory using the same method. These measurements may be useful for assessing kidney diseases. Finally, the dose of radiation exposure on CT was higher compared with radio-isotope examinations, for example, ^99m^Tc-DTPA and ^99m^Tc-MAG3.^18^ However, we suggest that CT provides detailed anatomical information before transplantation surgery compared with other examinations. Thus, in limited cases, such as in renal transplantation donors, CT is a useful technique and should be used.

## Conclusions

Dynamic CT enables calculation of CT-ERPF using our proposed method and is a feasible technique in renal transplantation donors.

## Figures and Tables

**Table1:** Processing time for setting ROIs in the renal cortex

Case number	Manual ROI (hours)	Automatic processing+manual correction (hours)		
		Automatic processing	Manual correction	Total time
11	16.0	0.0240	1.19	1.21
12	24.0	0.0238	1.65	1.68
17	24.0	0.0236	1.33	1.35
32	16.0	0.0250	0.75	0.77
33	16.0	0.0246	0.96	0.98
40	13.0	0.0230	1.24	1.26
42	12.0	0.0260	1.22	1.24
46	20.0	0.0250	1.76	1.79
47	14.0	0.0253	2.57	2.59
Average	16.7	0.0245	1.34	1.36

The required time to set ROIs in the renal cortex part manually and automatically on nine cases. Manual ROIs were confirmed by a radiologist who was engaged in Fujita Health University.
